# Germ cell development in the postnatal testis: the key to prevent malignancy in cryptorchidism?

**DOI:** 10.3389/fendo.2012.00176

**Published:** 2013-01-03

**Authors:** John M. Hutson, Ruili Li, Bridget R. Southwell, Bodil L. Petersen, Jorgen Thorup, Dina Cortes

**Affiliations:** ^1^Department of Urology, Royal Children’s HospitalParkville, VIC, Australia; ^2^F Douglas Stephens, Surgical Research Group, Murdoch Childrens Research InstituteMelbourne, VIC, Australia; ^3^Department of Pathology, University of CopenhagenCopenhagen, Denmark; ^4^Department of Paediatric Surgery and Pathology, Rigshospitalet, Faculty of Health Science, University of CopenhagenCopenhagen, Denmark; ^5^Department of Paediatrics, Hvidovre Hospital, Faculty of Health Science, University of CopenhagenCopenhagen, Denmark

**Keywords:** germ cell, gonocyte, spermatogium, testis, cryptorchidism, orchidopexy

## Abstract

To permit normal postnatal germ cell development, the mammalian testis undergoes a complex, multi-staged process of descent to the scrotum. Failure of any part of this process leads to congenital cryptorchidism, wherein the malpositioned testis finds itself at the wrong temperature after birth, which leads to secondary germ cell loss and later infertility and risk of cancer. Recent studies suggest that neonatal gonocytes transform into the putative spermatogenic stem cells between 3 and 9 months, and this initial postnatal step is deranged in cryptorchid testes. In addition, it is thought the abnormality high temperature may also impair apoptosis of remaining gonocytes, allowing some to persist to become the possible source of carcinoma *in situ* and malignancy after puberty. The biology of postnatal germ cell development is of intense interest, as it is likely to be the key to the optimal timing for orchidopexy.

## INTRODUCTION

The right way to treat undescended testes (UDT) remains to be solved. Although much is known about the development of the testes and sperm cells, there is a missing link in our knowledge. It is not known when and how the progenitors of germ cells transform from neonatal gonocytes into spermatogonia or spermatic stem cells. Recent research suggests that a critical stage of sperm cell development may occur in the early postnatal period. Germ cell development has been studied extensively in the fetus and at puberty, but not in this postnatal window. We will propose in this review that knowledge about early postnatal germ cell development is crucial for the optimal management of UDT.

Undescended testes, or cryptorchidism, is a major problem occurring in about 5% of neonatal boys, with surgery to pull the testes down to the scrotum (orchidopexy), currently recommended between 6 and 12–18 months (Ritzen et al., 2007; Hutson et al., 2010). It has been previously shown in numerous studies that cryptorchidism in the past was associated with a 30–60% risk of infertility or lack of germ cells, and a 5- to 10-fold increase in testicular malignancy. Crucial steps in maturation of gonocytes into the sperm cell lineage and of sperm cell development could be occurring in this early postnatal period and could be dependent on low temperature present in the scrotum but lacking in UDT. In addition, massive cell death should occur in non-maturing gonocytes, clearing the testis of any undifferentiated, pluripotential neonatal germ cells, as persisting cells could go on to undergo cell division and become cancerous. The timing of surgery for orchidopexy aims to prevent these problems but lacks scientific evidence. In this review, we examine the evidence for early postnatal germ cell development to determine what controls these steps and also to examine the evidence for early orchidopexy to try and prevent abnormal germ cell development.

## NORMAL EMBRYOLOGY AND POSTNATAL DEVELOPMENT IN HUMANS

**FIGURE 1:**
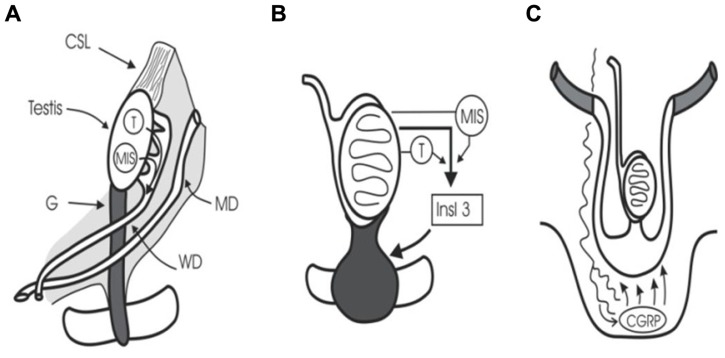
**Testicular descent**. **(A)** Cranial suspensory ligament (CSL) and gubernaculum (G) hold the testis. Testosterone (T) and MIS/AMH act on Wolffian duct (WD) and Müllerian duct (MD). **(B)** Insulin 3 (Insl3) causes the gubernacular swelling reaction that holds the testis near the groin during midgestational growth (8–15 weeks). **(C)** Calcitonin gene-related peptide (CGRP) released by the genitofemoral nerve, and under the control of testosterone, steers the migrating gubernaculum to the scrotum.

At 5 weeks of gestation, embryonic gonocytes migrate from the umbilical stalk into the ambisexual gonad. Human sexual development then begins at 7–8 weeks’ gestation when the SRY (sex-determining region Y) gene initiates testicular differentiation with Sertoli cell development (Sinclair, 1994). Cords of Sertoli cells surround the newly arrived germ cells and the interstitial cells form Leydig cells and peritubular myoid cells. Müllerian-inhibiting substance (or anti-Müllerian hormone) (MIS/AMH) and testosterone synthesis by Sertoli cells and Leydig cells, respectively, trigger regression of Müllerian ducts and preservation of Wolffian ducts which form epididymis, vas, and seminal vesicles (Wilson et al., 1981; Lee and Donahoe, 1993; **Figure 1**). The intra-abdominal fetal testis descends to the scrotum in a complex, two-stage process that in humans is complete at birth, while in rodents the second phase occurs in the first week after birth (Hutson et al., 1997). The regulation of this two-stage process is not the subject of this review, but interested readers should see some recent reviews (Ivell and Hartung, 2003; Adham and Agoulnik, 2004; Amann and Veeramachaneni, 2007; Bay et al., 2011).

**FIGURE 2:**
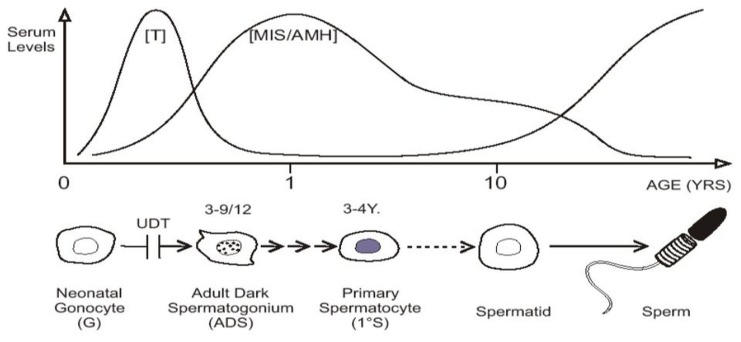
**“Mini-puberty,” with testosterone (T) and MIS/AMH secreted after birth**. Neonatal gonocytes mature into adult dark spermatogonia between 3 and 9 months of age, and primary spermatocytes form about 3–4 years. After a period of quiescence, spermatids form about 10 years of age, with the onset on spermatogenesis. Undescended testis (UDT) interferes with the first step.

In baby boys, the temperature of the intra-scrotal testes drops to 33°C at birth, with all the testicular enzyme systems readjusting presumably within a few weeks to this new environment (Zorgniotti, 1991). Between 2 and 4 months of age pituitary gonadotropins stimulate a sudden increase in testosterone production which peaks at about 3–6 months, before it wanes and becomes almost negligible until the onset of puberty. This brief surge of gonadotropins and androgens is known as “mini-puberty” (Job et al., 1988; Hadziselimovic and Zivkovic, 2007). As androgen levels subside there is a surge in production of MIS/AMH by Sertoli cells, which peaks at 6–12 months, but remains high then until puberty when serum levels fall (Baker et al., 1990; Yamanaka et al., 1991; Aksglaede et al., 2010; **Figure 2**).

## ABNORMAL POSTNATAL DEVELOPMENT IN UDT IN HUMANS

Failure of the first or second phase of testicular descent leads to congenital cryptorchidism. The causes of UDT remain largely unknown with some authors suggesting a primary testicular hormonal malfunction (Heyns and Hutson, 1995), while others have suggested a secondary hormonal malfunction, such a hypothalamic–pituitary axis deficiency (Hadziselimovic and Zivkovic, 2007) or a placental failure to produce chorionic gonadotropin (Heyns and Hutson, 1995). Based on twin studies, it has also been suggested that risk factors should be sought in the intrauterine environment and the maternal genes. However, another possibility first suggested in the eighteenth and nineteenth centuries, was that there was an anatomical defect in the mechanism of descent. With the establishment of endocrinology in the early twentieth century, these anatomical concepts were mostly replaced with explanations of hormonal dysfunction, such as described above (Heyns and Hutson, 1995). However, our own research over the last 25 years, along with others, has suggested that anatomical faults may be a common cause of cryptorchidism (Backhouse, 1964). Cryptorchidism is associated with other congenital abnormalities in less than 20% of the cases, but when present then most are often related to abnormalities of the midline and the caudal development field of the body (Cortes, 1998; Jensen et al., 2010; Thorup et al., 2010). Two key observations in favor of this view are the failure to document convincing primary hormonal defects, as well as the realization that the second phase of descent is a very complex anatomical process, that is likely to be prone to subtle anomalies (Amann and Veeramachaneni, 2007; Thorup et al., 2012). This is further supported by the fact that the anomaly most often occurs unilaterally, and in cases where associated Wolffian duct and/or ureteric bud malformations are present, these are predominantly found ipsilaterally (Cortes et al., 1998a).

If something goes wrong with migration of the gubernaculum from the external inguinal ring in the groin across the pubis and into the scrotum, the initially normal postnatal testis is retained deep to the inguinal fat pad, which is an effective insulator, keeping the UDT at 34–37°C (Mieusset et al., 1993), rather than the normal scrotal temperature of 33°C, and this is believed to trigger the progressive postnatal testicular dysfunction, as the postnatal testis function is only optimal at 33°C (Zorgniotti, 1991). The exquisite temperature sensitivity of the testis has been well documented for a long time, but is currently not a fashionable area of research in this era of molecular biology. However, the phenomenon has been solidly proven despite the genetic regulation of it not being fully described. This abnormal postnatal development in the testis also leads to inhibited postnatal androgen production (Raivio et al., 2003) and MIS/AMH production at 6–12 months of age (Yamanaka et al., 1991).

The net effect of cryptorchidism is germ cell loss, leading to infertility (Mengel et al., 1974; Hadziselimovic, 1985). This has been well described in many long-term outcome studies in men with a previous history of cryptorchidism in childhood (Cortes, 1998; Gracia et al., 2000; Cortes et al., 2001; Vincardi et al., 2001). However, the cause for this germ cell loss and its timing have remained elusive until relatively recently. In the 1950s, orchidopexy was recommended in boys aged 10–15 years if the cryptorchid testes failed to descend spontaneously into the scrotum at the onset of puberty (Gross and Jewett, 1956; Vincardi et al., 2001). This recommendation was based on the clear observation that a significant number of cryptorchid testes descended spontaneously at puberty. In addition, it was assumed that germ cell development was totally quiescent in childhood. Indeed, it was thought that the testis was effectively in “suspended animation” until the onset of spermatogenesis in early puberty.

Current long-term follow-up studies of men with a previous history of cryptorchidism show a 5- to 10-fold increase in malignancy risk (Whitaker, 1988; Wood and Elder, 2009). However, it is easy to forget that because of the very long lag-time (i.e., 30–40 years) between intervention and measurement of the outcome, in this case development of testicular malignancy, that the age of orchidopexy in most of these studies in the current literature is about 5–15 years of age. This has led to the widespread view in adult endocrinology and oncology that orchidopexy is unable to prevent development of cancer in men (Gracia et al., 2000). However, the age for orchidopexy is now much younger, and it remains to be seen whether this will alter outcomes in adulthood. Recent studies actually indicate that operation for cryptorchidism at a young age lowers the risk of testicular cancer in adulthood (Pettersson et al., 2007).

**FIGURE 3:**
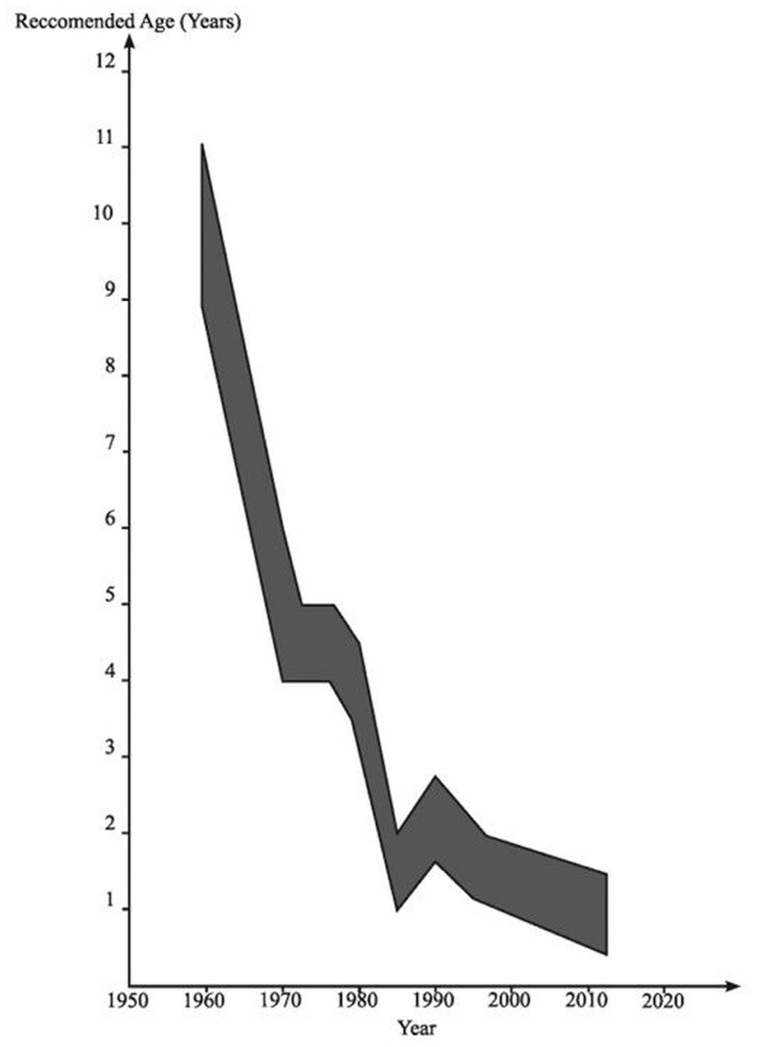
**The age at which orchidopexy has been advocated since the 1950s**. Reproduced with modifications from Hutson and Beasley (1992).

Meanwhile, in pediatrics, it first became apparent in the 1970s that degeneration of the UDT was occurring in early childhood (Mengel et al., 1974). Initially it was noted that there was macroscopic atrophy of the testis in early primary school years leading to the view that maybe orchidopexy should be done in 5- to 6-year-old boys rather than at 10–15 years, based on the assumption that this might prevent the obviously visible development of atrophy. During the 1970s and early 1980s there was histological evidence accumulating of degeneration visible in boys of 2 years of age with UDT, leading to the recommendation in pediatric surgery that orchidopexy might be optimally done in 2-year-old boys (Hadziselimovic et al., 1975). It was then appreciated that there were signs of early degeneration in the testis on electron microscopy at about 12 months of age (Hadziselimovic, 1985; **Figure 3**).

Furthermore, it was reported that all UDT harbored germ cells at the time of birth, but the number of germ cells was decreased in about one-fourth of cryptorchid newborns (Cortes, 1998). Lack of germ cells has been reported from 12, and especially from 18 months of age, and therefore surgery has been recommended before 12 or 18 months of age (Ritzen et al., 2007).

Recent studies have implicated the very first phases (i.e., in the first year) of postnatal germ cell development in the etiology of the subsequent infertility and risk of malignancy. However, we first need to describe normal germ cell development so that the effects of cryptorchidism can be understood in context.

## NORMAL GERM CELL DEVELOPMENT

Around 4–6 weeks of gestation within the embryonic urogenital ridge the primitive gonad forms on the anteromedial surface of the middle kidney or mesonephros. Primordial germ cells form from endodermal cells in the caudal edge of the yolk sac stalk, and then migrate into the embryonic celom around the wall of the midgut and into the urogenital ridge, eventually colonizing the ambisexual gonad and differentiating into gonocytes. By 22 weeks of gestation, these primitive germ cells mature into fetal spermatogonia (Hadziselimovic, 1983). Mesenchymal cells in the 7- to 8-week ambisexual gonad surrounding the arriving germ cells form the Sertoli cells with the onset of sexual differentiation to create the testicular cords, inside a basement membrane. Outside the cords some mesenchymal cells form Leydig cells and begin hormone synthesis to produce androgen and insulin-like hormone 3 (INSL3), while others form into myoepithelial cells around the cords.

At birth, the fetal spermatogonia or neonatal gonocytes are located in the center of the spermatic cords. Throughout childhood it appears that the germ cells remained in the center of the cords, and seemed to be in “hibernation” until the onset of spermatogenesis at puberty, as there was little histological change in their appearance or location. However, in recent years, it became apparent that by 3–4 years of age the cell in the center of the testicular cords is a primary spermatocyte, rather than a neonatal gonocyte (Hadziselimovic, 1983).

Recent studies show that the neonatal gonocyte migrates between the Sertoli cells to the periphery of the cord between 3 and 9 months of age, or 2 and 6 days of age in mice (Drumond et al., 2001; Huff et al., 2001b), where it comes in contact with the basement membrane. These factors trigger transformation into type-A spermatogonia, which line the basement membrane, displacing the adjacent Sertoli cells. The regulatory factors involved in this transformation are mostly unknown (see below), although platelet-derived growth factors (PDGF) B and D, and also PDGF receptor-beta (PDGFR-β) have been implicated (Basciani et al., 2008, 2010). Inhibition of PDGFR-β tyrosine kinase activity in the first week postnatally in a mouse causes a severe reduction in the proliferation of gonocytes and increases their apoptosis (Basciani et al., 2008, 2010). Type-A spermatogonia mature into type-B spermatogonia, then migrate back into the center of cord again by 3–4 years, to become primary spermatocytes.

Alongside these studies of early postnatal germ cell development have been studies searching for the potential stem cell of spermatogenesis, with the aim of using these stem cells for recolonizing the infertile testis (Brinster, 2007; Waheeb and Hofmann, 2011). Based on morphological criteria, human spermatogonia have been classified as dark type-A (Ad), pale type-A (Ap), and type-B. Ad-spermatogonia have nuclei showing a homogeneous, dark stainable chromatin and one or more clear cavities of vacuole like appearance (Clermont, 1963). The type-A spermatogonium has become the likely stem cell of spermatogenesis (de Rooij and Russell, 2000; Drumond et al., 2001; Biers and Malone, 2010). Interestingly not all neonatal gonocytes transform into type-A spermatogonia, so that by 1–2 years of age the total germ cell number is less than half that at birth (Hadziselimovic, 1983). The remaining gonocytes are thought to undergo apoptosis, or programed cell death, so that by 2 years of age there are none left in the testis (Huff et al., 2001b). This process can be seen readily in the postnatal rat testis, as signs of apoptosis are rare until day 3, but by day 4 analysis shows that one-third of them are lost (Roosen-Runge and Leik, 2000).

## THE SPERMATOGENIC STEM CELL

As neonatal gonocytes seem to differentiate into unipotent stem cells for subsequent spermatogenesis, their change in appearance, location within the cord, and function suggest dramatic changes in germ cellular physiology. These changes are regulated by a precisely coordinated expression of key proteins, some of which are known.

In early embryos, germ cells and pluripotent embryonic stem cells can form any type of cell. However, as development proceeds, the ability of germ cells to form different cells becomes restricted. In testes a subpopulation of germ cells retain the ability to differentiate, but only as unipotent spermatogenic stem cell (SSCs; Hofmann et al., 2005).

**FIGURE 4:**
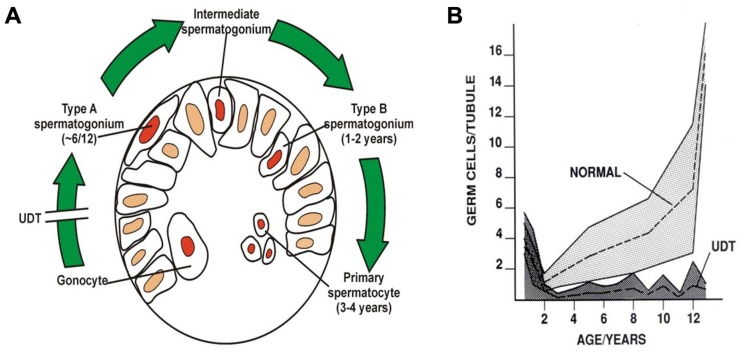
**Postnatal germ cell development in humans**. **(A)** Gonocytes migrate from the center of the cords to the basement membrane around 6 months, and become type-A spermatogonia. By 3–4 years of age the center of the cords becomes recolonized with primary spermatocytes. **(B)** Numbers of germ cells/tubule in a normal testis and undescended testis (UDT) relative to age in years. Note the normal fall in total numbers between birth and 2 years, but failure of this to recover in UDT. The shaded area shows the normal range and the dotted lines show the average numbers.

Huckins and Oakberg have proposed a widely accepted model for spermatogenic development which is useful to describe here (Huckins, 1971; Oakberg, 1971; de Rooij and Russell, 2000). In this model, single type-A spermatogonia are the putative SSCs, which can self-renew, while paired type-A spermatogonia are differentiating paired daughter cells connected by an intercellular bridge. Paired type-A spermatogonia divide into chains of aligned cells, which then become type A_1_, A_2_, A_3_, and then A_4_ spermatogonia. The latter cells (A_4_) divide to form intermediate spermatogonia and the type-B spermatogonia. Type-B cells then divide to form primary spermatocytes that enter meiosis (**Figure 4**). All these steps are regulated by growth factors from Sertoli and possibly the peritubular myoid cells (Skinner, 1991; Jegou, 1993). A recent morphological study (Drumond et al., 2001) showed that postnatal development of type-A spermatogonia may occur more rapidly than in mature spermatogenesis.

**FIGURE 5:**
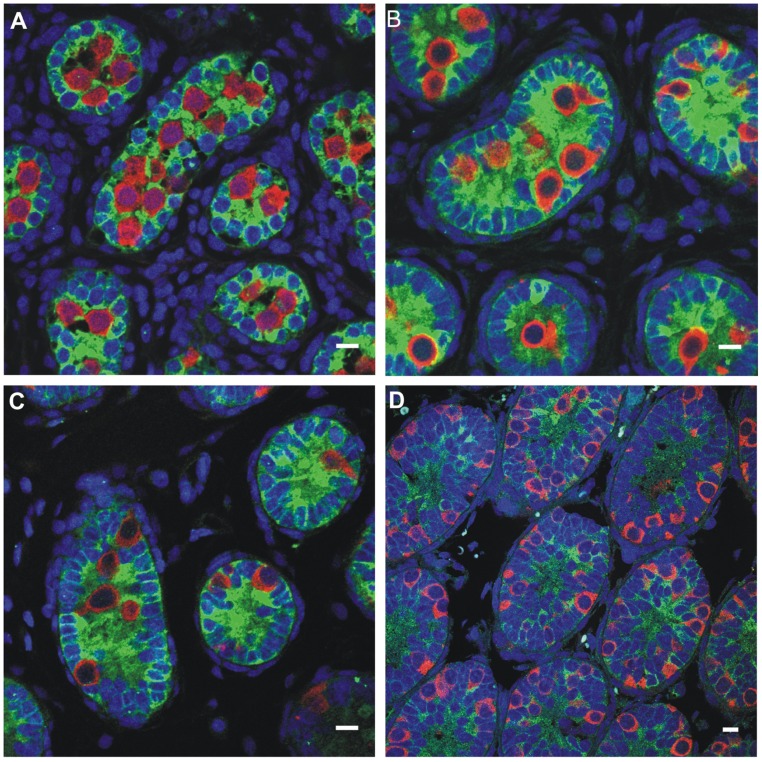
**Germ cell development in the first week postnatally in the rat, showing the germ cells labeled with MVH (mouse homolog of Drosophila Vasa), and the Sertoli cells labeled with MIS/AMH (bar = 10 μm)**. **(A)** Day 0, **(B)** Day 4, **(C)** Day 6, **(D)** Day 10.

The type-A spermatogonia and SSCs have been shown recently to express a range of different markers (**Figure 5**). Undifferentiated SSCs express the integrins β-1 and α-6 (Shinohara et al., 1999), and the receptors for glial-derived neurotrophic factor (GDNF), such as GDNF family receptor alpha-1 (GFRα-1) and receptor tyrosine kinase (RET) (de Rooij and Russell, 2000). In addition undifferentiated SSCs express Zbtb-16 (previously known as Plzf; Buaas et al., 2004; Costoya et al., 2004). Once SSCs start to differentiate, they begin expressing a range of different markers, including c-Kit, the receptor for stem cell factor (Besmer et al., 1993; Yoshida et al., 2006). They also express Sohlh1 and Sohlh2, as well as neurogenin 3 (Ballow et al., 2006; Filipponi et al., 2007; Hao et al., 2008). As can be seen from this brief overview, most of what we know about SSCs comes from studies of adult spermatogenesis or stem cell research, rather than a direct study of the type-A spermatogonium in the postnatal testis.

## ABNORMAL GERM CELL DEVELOPMENT

**FIGURE 6:**
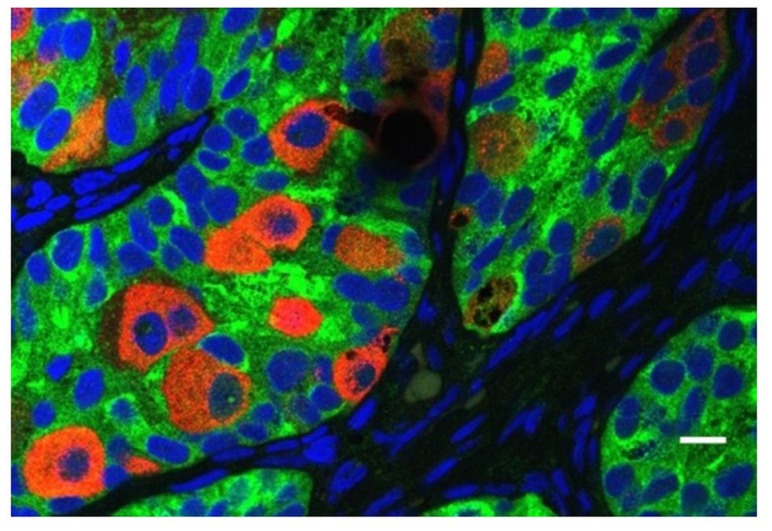
**Abnormal persistence of gonocytes beyond 6 months of age in a boy with cryptorchidism**. The germ cells are labeled with MVH (mouse homolog of Drosophila Vasa), and the Sertoli cells are labeled with MIS/AMH (bar = 10 μm).

All cryptorchid testes of newborns contain germ cells, although in some testes the number is impaired compared to normal. So significant deranged germ cell development in cryptorchid testes probably begins postnatally, except in the dysgenetic testis in disorders of sex development (DSD). The first step to fail is transformation of gonocytes into type-A spermatogonia, which is delayed or interrupted. Evidence for this disruption is found in the persistence of large numbers of gonocytes in the center of the testicular cords well beyond 6 months of age (**Figure 6**), and a decreased number of type-A spermatogonia (Hadziselimovic et al., 1986; Huff et al., 1989, 1991). After the first year or so the number of spermatogonia decrease, while those that remain are gonocytes with bizarre nuclei (Hadziselimovic, 1983). By 3–4 years of age the abnormality has become even more obvious, with failure of primary spermatocytes to appear (Huff et al., 1989).

The abnormally high temperature of the cryptorchid human testis is considered by most authors to be the cause of this germ cell maldevelopment. Considerable studies have been performed in various animal models showing that heat stress leads to a combination of both indirect and direct effects on the germ cells, causing impaired transformation and maturation as well as inhibited apoptosis. This thermal injury is mediated by reactive oxygen species and certain heat-shock proteins, which damage the germ cells as well as Sertoli cells (Setchell, 1998; Zini et al., 1999; Ivell and Hartung, 2003).

Defective transformation of gonocytes into type-A dark spermatogonia between 3 and 9 months of age correlates with abnormal sperm counts after puberty (Hadziselimovic and Herzog, 2001a; Huff et al., 2001a). In cases of no adult dark spermatogonia, the age of orchidopexy has not affected a poor result for spermatogenesis in adulthood, at least so far (Hadziselimovic and Herzog, 2001b). The degree of risk of subsequent infertility can be quantitated, as it is almost inevitable if there are fewer than 1% of the number of spermatogonia per tubular cross-section, seen in biopsies from age-matched controls (Cortes et al., 1996). Under normal physiological circumstances, the surge of gonadotropins and testosterone, and possibly other as yet unknown hormones, occurs at 2–3 months of postnatal age. This hormonal surge stimulates the gonocytes in the testis to develop into adult dark spermatogonia. In cryptorchidism there has been reported evidence for a beneficial role of adjuvant gonadotropin-releasing hormone to improve fertility in UDT (Biers and Malone, 2010). It has been questioned as a general recommendation (Hutson, 2010). Recently there have been described different groups of cryptorchid boys, and if the patients at the time of surgery have a decreased number of germ cells and/or inhibin B and no compensatory increase in gonadotropins, then supplementary hormonal treatment may be indicated after early surgery for UDT (Thorup et al., 2012). It has been suggested that antisperm antibodies may be involved in the germ cell loss in cryptorchidism, but there is no evidence for postpubertal antibodies after previous surgery in childhood for cryptorchidism (Mirilas et al., 2003). All these studies suggest that the postpubertal poor fertility is likely to be caused by an abnormally small pool of SSCs, the type- A spermatogonia.

The risk of cancer after cryptorchidism in infancy is less easily correlated with abnormal germ cell development after birth. It has been well described that the risk of developing a testicular germ cell cancer is about 5- to 10-fold higher in men with a previous history of cryptorchidism compared with those with descended testis in childhood (Chilvers et al., 1986; Giwercman et al., 1987; Moller et al., 1998; Wood and Elder, 2009). Testicular germ cell tumors are extremely common, affecting 1% of young men (Rorth et al., 2000).

The common precursor of these tumors is the premalignant carcinoma *in situ* (CIS) cell, which is proposed to be a fetal germ cell that failed to differentiate, and remains, dormant in the testis until after puberty (Skakkebaek et al., 1982). The evidence supporting this view comes from the many similarities between CIS cells and fetal gonocytes, which share many markers and morphological characteristics (Holstein and Korner, 1974; Skakkebaek et al., 1982; Rorth et al., 2000; Rajpert-De Meyts et al., 2003). CIS cells may well originate during fetal development in DSD, where the germ cell physiology is deranged by the underlying genetic anomaly. By contrast, this seems less plausible in cryptorchidism if the maldescent is caused by extraneous mechanical defects. In cryptorchidism, therefore, the CIS cell may arise from the neonatal gonocytes, which are abnormally abundant. The gonocytes have pluripotent stem cell properties, which if they persist until after puberty, with or without mutation, may develop into cancer.

Carcinoma *in situ* cells are more likely to be present where there are specific genetic defects, and there may be developmental arrest (Skakkebaek et al., 1987, 1998; Honecker et al., 2004). Expression profiles of CIS cells and embryonic stem cells are very similar, which supports the view of CIS being a multipotent stem cell (Almstrup et al., 2004). A unifying hypothesis has been proposed to link testicular maldevelopment, testicular cancer, low sperm counts, and cryptorchidism, known as the testicular dysgenesis syndrome (TDS; Asklund et al., 2004).

Carcinoma *in situ* is more common in men with previous cryptorchidism, and is documented in 2–3% of adult patients (Giwercman et al., 1987, 1989). However, the prevalence of CIS is higher in those men with macroscopic testicular atrophy (Harland et al., 1998) or those with bilateral cryptorchidism (Swerdlow et al., 1997). Where cryptorchidism is unilateral, the contralateral descended testis has a slightly increased risk of cancer (Forman et al., 1994). Not surprisingly, cancer risk is also increased where testes were intra-abdominal, the genitalia were abnormal or there was a known abnormal karyotype (Cortes et al., 1999).

**FIGURE 7:**
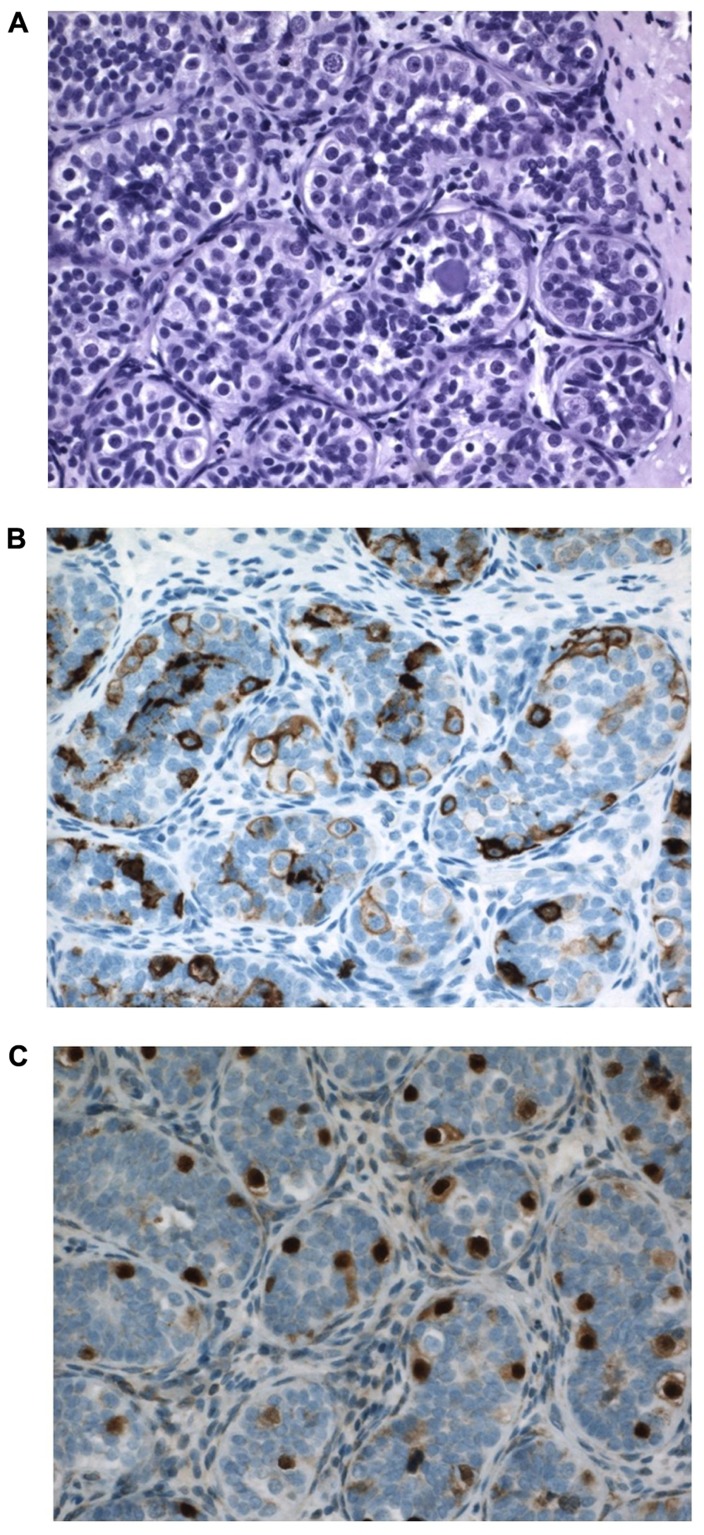
**Testicular tissue from a 12-month-old boy with bilateral cryptorchidism**. Carcinoma *in situ* (CIS) is suspected. **(A)** H&E staining: Sertoli cells are normal and the germ cell number is at the upper range for age. Some germ cells have two nuclei, and some nuclei have irregular morphology and some nucleolus. A microlith is clearly seen within a tubule. **(B)** Strong PLAP expression seen in all germ cells. **(C)** OCT4 expression in germ cells also in the periphery of tubules, which is not often seen so impressively in most cryptorchid testes at a similar age.

In a pediatric collective series of six studies, the risk of CIS in 2800 boys with cryptorchidism was 0.36% only (Cortes, 1998). When compared to higher incidence data from adults these pediatric figures support the hypothesis that the characteristics of CIS cells and later testicular cancer develops over time, and are not present from fetal life. Generally CIS in pediatric series is described in later childhood (Cortes et al., 1999). However, in this paper we present a 12-month-old boy with suspected CIS in bilateral cryptorchid testes (**Figure 7**). It has to be emphasized that positive immunohistochemical markers indicating CIS in the adult testis do not have such predictive value when applied to the childhood cryptorchid testis. Positive staining by such markers of persistent fetal germ cells is seen in almost half of the cryptorchid testes of boys younger than 5 years (Thorup et al., 2012). Presence of placental alkaline phosphatase (PLAP)-positive immunohistochemical stained gonocytes in bilateral cryptorchid testes of infant boys indicates the ability of delayed germ cell transformation and a preserved good fertility potential (Cortes et al., 2012). Further research in this field is therefore needed.

It has been suggested that testicular biopsy itself at the time of orchidopexy is a risk factor for postpubertal cancer (Swerdlow et al., 1997). In the review by Swerdlow et al. (1997) only 9% of the operated testes were subjected to biopsy, consistent with a possible selection bias by the surgeon, who may have only picked the more atrophic testes for analysis. This view is supported by a large study of 830 cryptorchid patients having routine biopsy, where there was no correlation between the biopsy itself and subsequent malignancy (Moller et al., 1998).

Some authors have suggested that adolescents with a previous history of cryptorchidism and orchidopexy should be offered a biopsy to identify if CIS is present, before invasion has occurred (Giwercman et al., 1989). Alternative screening methods such as reliable blood or semen analysis are not yet available, although testicular ultrasonography may have a place (Holm et al., 2001). Ultrasonography shows a relationship between microlithiasis and CIS, but the significance of this is uncertain (Lenz et al., 1996), particularly as more recent studies of testicular microlithiasis show that it is quite common in normal adolescents (Goede et al., 2009). CIS is also associated in some patients with cryptorchidism with multinucleated germ cells, consistent with aberrant germ cell development (Cortes et al., 2003).

There is inconclusive evidence that an early age of surgery leads to a reduction in risk of cancer (Pike et al., 1986; Swerdlow et al., 1997; Wood and Elder, 2009). However, because of the long lag-time between treatment and outcome, the age at surgery in these follow-up studies averaged more than 2 years old. In the context of the proposal, that early germ cell development is the key, these long-term results are consistent with either no effect of age at all, or with the suggestion that lack of neonatal gonocyte transformation and impaired apoptosis predisposes to malignancy.

One of the problems that has bedeviled the relationship between cryptorchidism and malignancy is the lack of an animal model, as rodents with UDT do not develop testicular tumors. Another problem is that evidence is accumulating that there are some testes that become undescended after birth, and that this group, now known as acquired cryptorchidism, have a different risk of malignancy compared with congenital UDT.

## ACQUIRED CRYPTORCHIDISM

After birth, the distance from the inguinal canal to the scrotum increases from 4–5 to 8–10 cm in early adolescence (Smith et al., 1989). This means that the spermatic cord must double in length between birth and puberty (Hutson et al., 2010). We have previously proposed that failure of this elongation is the cause of ascending testes or acquired UDT (Hutson and Hasthorpe, 2005), and this is the likely explanation for many children still having orchidopexy at 5–10 years, despite recommendations for surgery in infancy (Donaldson et al., 1996).

Realization that there are two types of UDT has triggered a re-evaluation of the cause and effect of UDT. First, it is becoming apparent that men who had untreated, acquired UDT have no increased cancer risk, but still have impaired fertility (Cortes et al., 1998b). Secondly, it means that current long-term follow-up studies in adults are likely to contain a mixture of patients with both congenital and acquired UDT (Bonney et al., 2008), making their interpretation difficult.

**FIGURE 8:**
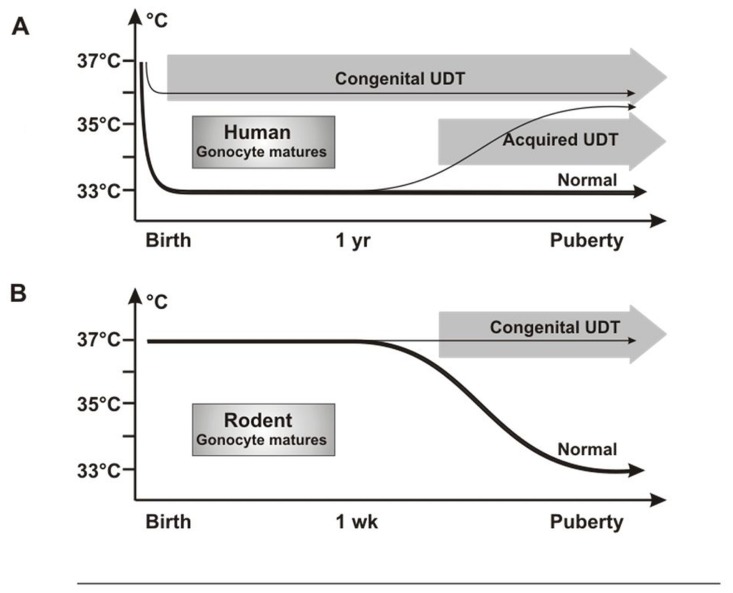
**Testicular temperature in humans and rodents**. **(A)** Human gonocytes mature in the first year when the testicular temperature is 33°C. Congenital UDT have abnormal temperature early, which interferes with gonocyte development. By contrast, acquired UDT only interferes with subsequent survival of stem cells. **(B)** In rodents, gonocytes mature before a change in temperature occurs, so that gonocyte development is unaffected, similar to acquired UDT in humans.

Investigators have puzzled over why UDT in rodents caused infertility but not cancer, as in humans. We propose that this is the result of differences in timing in when the testis reaches the low-temperature scrotal environment. In humans, the testis is at 33°C from birth, so that congenital UDT has the potential to damage neonatal gonocytes by high temperature. By contrast, rodent testes only reach the scrotum after 12 days of age (Bergh, 1991), which is well after normal gonocyte transformation and/or apoptosis is complete (**Figure 8**).

Undescended testis in rodents, therefore, is actually a model for acquired UDT in humans, with high temperature damaging the SSCs, but with no cancer risk as the gonocytes will have developed or disappeared normally. Once this key difference in timing of testicular descent is understood, extrapolation between rodent models and the human situation is straightforward, as other key processes are the same. Both species groups have a postnatal “mini-puberty” at 3–6 months in humans and 2–4 days in mice, when there is burst of testicular hormones and cytokines which are likely to be involved in gonocyte development. In addition, gonocyte migration from the center of the cords to the basement membrane to form SSCs is similar in both timing and general morphology.

A temperature difference between undescended and normally descended testes in rats is not present until after 12 days of age (Zorgniotti, 1991), which is well after the gonocytes should have transformed normally into SSCs or have undergone apoptosis. If excess persisting gonocytes are the cause of CIS and cancer in cryptorchidism, then none will be present in the testis when the temperature becomes abnormal in rodent UDT, consistent with the lack of cancer in these models. However, the high temperature of the UDT will still damage SSCs leading to subsequent infertility (**Figure 8**).

## CONCLUDING REMARKS

All the evidence available points to early germ cell maturation being the “missing link” in the disconnection between the timing of orchidopexy and the subsequent risks of both infertility and malignancy. This suggests that infants getting really early operation may have significantly improved outcomes, although this remains hypothetical. Furthermore, in some cases supplementary hormonal treatment may be needed to achieve the normal transformation to adult dark spermatogonia.

The key role of heat in interfering with the signaling that controls both gonocyte transformation and/or apoptosis needs to be reinvestigated fully, as it is likely to lead to changes in clinical management of congenital cryptorchidism.

The recognition of acquired UDT as a real clinical entity has not only informed our understanding of the possible management in children, but also provided an insight into why rodent animal models of cryptorchidism do not demonstrate testicular cancer. In both circumstances, the abnormal temperature affects the testis well after gonocyte maturation or apoptosis is complete, so that secondary heat shock depresses stem cell function alone, leading to infertility.

The next few years should help to shed light on these new developments further, so that we can determine the optimal treatment, which may include a differentiated treatment strategy for different groups of cryptorchid boys. Equally, the risk of infertility and testicular cancer may not be the same in all boys with cryptorchidism.

## Conflict of Interest Statement

The authors declare that the research was conducted in the absence of any commercial or financial relationships that could be construed as a potential conflict of interest.
